# Bandgap Narrowing of BaTiO_3_-Based Ferroelectric Oxides through Cobalt Doping for Photovoltaic Applications

**DOI:** 10.3390/ma16247528

**Published:** 2023-12-06

**Authors:** Mansour K. Gatasheh, Mohamed Saad Daoud, Hamoud Kassim

**Affiliations:** 1 Department of Biochemistry, College of Science, King Saud University, P.O. Box 2455, Riyadh 11451, Saudi Arabia; mgatasheh@ksu.edu.sa (M.K.G.); mdawood@ksu.edu.sa (M.S.D.); 2 Department of Physics & Astronomy, King Saud University, P.O. Box 2455, Riyadh 11451, Saudi Arabia

**Keywords:** photovoltaics, bandgap, oxygen vacancies, Raman modes, band structure, density of state

## Abstract

Following the finding of power conversion efficiency above the Shockley–Queisser limit in BaTiO_3_ (BTO) crystals, ferroelectric oxides have attracted scientific interest in ferroelectric photovoltaics (FPV). However, since ferroelectric oxides have a huge bandgap (>3 eV), progress in this sector is constrained. This paper proposes and demonstrates a new ferroelectric BaTi_1−x_Co_x_O_3_ powder (0 ≤ x ≤ 0.08), abbreviated as BTCx, that exhibited a bandgap decrease with increased Co content. Notably, changing the composition from x = 0.0 to 0.08 caused the system to show a bandgap drop from 3.24 to 2.42 eV. The ideal design with x = 0.08 displayed an abnormal PV response. Raman spectroscopy measurements were used to investigate the cause of the bandgap decrease, and density functional theory was used to interpret the analyzed results. According to our findings, Co^2+^ doping and oxygen octahedral distortions enhance bandgap reduction. This research sheds light on how bandgap tuning developed and laid the way for investigating novel low-bandgap ferroelectric materials for developing next-generation photovoltaic applications.

## 1. Introduction

The current fossil fuel supply is decreasing, causing research into renewable and alternative energy sources to be prioritized. Research into solar energy, the most plentiful renewable resource, has increased in popularity since the discovery of the photovoltaic (PV) effect [1,2,3]. The bandgap equivalent voltage limits the highest output voltage of commercially available conventional p-n-junction-based solar cells [4,5,6]. Above-bandgap photovoltage has been found in ferroelectric systems, making them a promising alternative PV material [7,8,9]. By isolating the charge carriers created by light, the electric field intrinsic to most ferroelectric materials may be switched on and off to regulate the voltage output [10,11,12]. Since it was shown that power conversion efficiency in BaTiO_3_ systems may surpass the Shockley–Queisser limit, interest in ferroelectric PV has increased [13]. Though various methods, including Co and Fe, co-doped Bi_3.25_Sm_0.75_TiO_12_ [14], Co and Fe co-doped Bi_3.25_Nd_0.75_TiO_12_ [15], PbTiO_3_ [16], and BiFeO_3_ [17], have been researched for the ferroelectric PV effect, the wide bandgap (>3 eV) found in these systems hampers their application potential. A ferroelectric system with a bandgap in the visible light absorption region is required to fully exploit its potential in PV systems. The bandgap in ABO_3_ perovskite is caused by the vacant d-orbital of the transition metal B-cations in the conduction band and the O-2p orbital in the valence band [18,19]. The oxygen anion has less electronegativity than the B-site cation in the d_0_ configuration, contributing to the large bandgap in oxide systems [20].

Ferroelectric perovskite polarization occurs due to the off-centering of d_0_ ions in the B site from the BO_6_ octahedral site or cations in the A-site octahedral site [21]. Incorporating a non-d_0_ transition metal into a material to reduce the bandgap might negatively impact its ferroelectricity and, therefore, its photovoltaic (PV) properties. To improve PV performance metrics, it is essential to build ferroelectric systems that exhibit both a low bandgap and high polarization. Specifically, inversion symmetry breaking is triggered in the prototype lead-free ferroelectric BTO system by distortion caused by the hybridization of the lowest unoccupied energy levels of Ti^4+^ ions and the oxygen ion [22,23]. N. V. Sarath et al. [23] observed that the manufactured ferroelectric (1 − x)BTO − xBi(Ni_2/3_Nb_1/3_)O_3_ (BTBNN) system exhibits a shift in bandgap from 3.1 to 2.4 eV with no polarization degradation. They linked the increase in PV to structural distortion.

The high polarization and huge bandgap (3.1 eV) result from a dipole caused by the off-centering of Ti^4+^ from the oxygen octahedra [24]. Bandgap reduction by B-site doping with cations with half-filled d-orbitals is conceivable, as has been shown in theoretical investigations of BaTiO_3_ (BTO) and PbTiO_3_ (PTO) [25,26]. However, it has been found that the polarization features of these systems are inhibited at room temperature when the bandgap is decreased [27,28]. This research reports the successful tuning of Co-doped BTO samples based on the PV device requirement. Co ions with partly filled d-orbitals might generate the defect level in the bandgap, allowing for absorption across the more extended wavelength range. It is worth noting that the bandgap of the synthesized system shifts from 3.24 to 2.42 eV. This paper uses Raman investigations to demonstrate a connection between qualitative structural distortion and bandgap energy. Our research elucidated a mechanism with far-reaching ramifications by determining the significance of local electrical structure in bandgap narrowing. New findings improve our understanding of cobalt-doped BaTiO_3_ and assist the design and engineering of materials for improved solar performance. The bandgap narrowing seen experimentally is corroborated by the density functional theory (DFT) finding, which shows the occurrence of Co-3d midcap states. This work mainly emphasizes the purposeful narrowing of the bandgap energy in cobalt-doped BaTiO_3_. Unlike typical bandgap engineering approaches, which can entail complicated material combinations or heterostructures, this research took a holistic approach by incorporating cobalt dopants into a single material. The ensuing lowering of the bandgap redefines the limitations of solar device design and operation, providing a more straightforward but highly effective approach to higher energy conversion efficiency.

## 2. Experimental Parameters

In this study, we used a solid-state reaction technique for synthesizing ceramics with the nominal BaTi_1−x_Co_x_O_3_ formula powders (0 ≤ x ≤ 0.08), abbreviated as BTCx. The ingredients used were BaCO_3_ (Sigma-Aldrich (Burlington, MA, USA), 99.99% purity), TiO_2_ (Sigma-Aldrich, 99.98% purity), and Co_3_O_4_ (Sigma-Aldrich, 99.98% purity). The raw materials were weighed and grained through high-energy ball milling for 10 h before being calcined at 1150 °C in a conventional furnace with a heating/cooling rate of 5 degrees Celsius per minute and a dwell time of 6 h. The calcined powders underwent ball milling again and sieving to obtain a uniform particle size. Powder X-ray diffraction (XRD) was performed using a Bruker D8 machine to characterize the phase structure of the calcined powders. The phase structure and estimated lattice parameters were all calculated using Full-Prof refinement software. We collected Raman spectra using a Wi-Tec micro-Raman spectrometer excited by an Nd: YAG laser at 532 nm and a CCD camera. The calcined powders were characterized optically at room temperature using a UV-2600 (UV-Vis spectrophotometer, Shimadzu) in the 200–800 nm wavelength range. Burai v1.3.1 software was employed to design the band structure and density of state for the cobalt-doped BaTiO_3_ sample.

## 3. Results and Discussion

### 3.1. XRD Analysis

**Figure 1:**
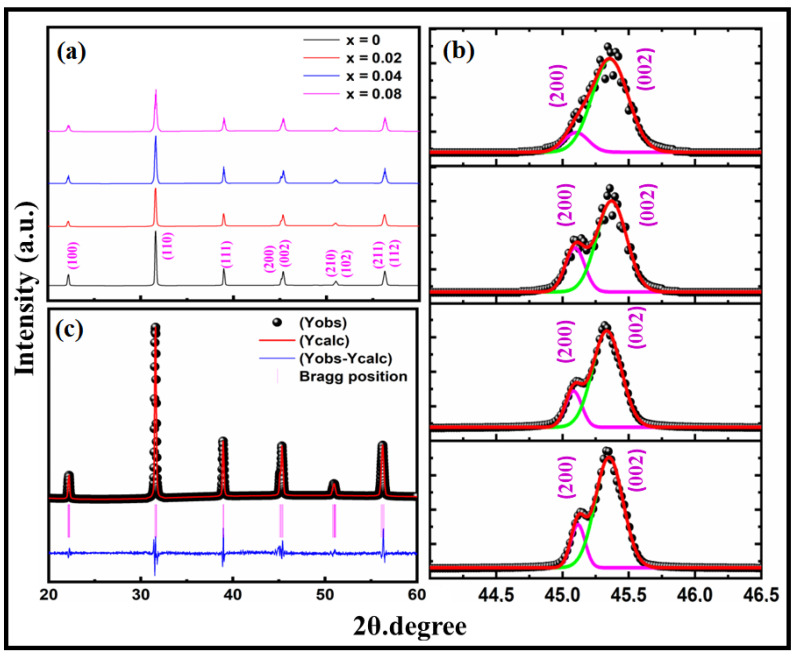
(**a**) X-ray diffraction of BTCx samples recorded at room temperature, (**b**) 200, 002 peaks fitting (black symbols represented the measured data, while the red cure represented the Gaussian fitting curve), and (**c**) XRD Rietveld refinement of the BTC0.04 sample, performed using Fullprof refinement.

**Figure 2:**
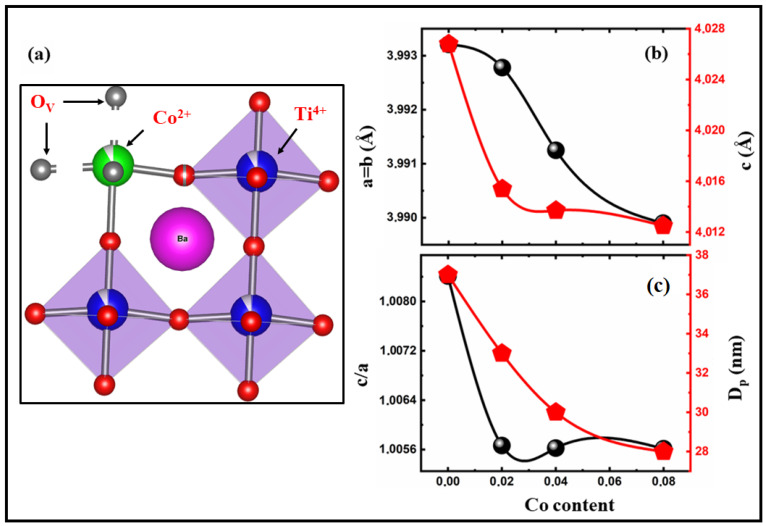
(**a**) Schematic structure diagram of the crystal structure of tetragonal Co-doped BT lattice Co^2+^ atom indicated by green color, oxygen vacancies (Vo) is indicated by silver color, Ti^4+^ atoms are indicated by blue color, Ba^2+^ atom is indicated by pink color, and oxygen atoms are indicated by red color (**b**) a representation of unit cell parameters (a = b parameter black curve, c parameter Red-curve), and (**c**) a representation of the tetragonality (c/a in black curve) and crystallite size (Dp in red curve) of the investigated samples with Co-doping-contents-doped BT lattice obtained from XRD refinement and Scherrer formula, respectively.

Figure 1a displays the XRD patterns of BTCx samples measured at room temperature. The decrease in the relative intensity of the fitted 002/200 peaks in Figure 1b and the existence of a single peak (111) at 2θ = 38.84° demonstrate that all samples had a single phase with a tetragonal structure and a P4mm space group matched with standard (JCPDS file number #83-1880) perovskite BaTiO_3_ ceramics [29]. XRD refinement is a crucial step in crystallography where the experimental X-ray diffraction data are used to refine the structural parameters of a crystal lattice model. The goal is to adjust the model’s parameters (such as atom positions, unit cell dimensions, thermal vibrations, etc.) to minimize the difference between the observed diffraction pattern and the pattern predicted by the model. This process ensures that the refined crystal structure accurately represents the arrangement of atoms within the crystal lattice. XRD refinement involves iterative calculations that minimize the discrepancy between the observed and calculated diffraction intensities. Various mathematical methods, such as least squares fitting, match the observed and calculated data. The refined parameters provide valuable information about the effect of Co ion doping on the crystal structure of BaTiO_3_ samples. Figure 1c shows the X-ray diffraction refinement estimates of the lattice parameters (a and c) shown graphically in Figure 2b. When the Co ions are introduced into the Ti^4+^ site, the lattice parameters (a and c) and tetragonality of the BT sample are shown to drop considerably. The host lattice is responsible for this reduction, including those of the Co ions in the BT. The ionic valences must also be taken into consideration. Specifically, the incorporation of Co in the Ti site is attributed to the difference in ionic radii between the doping and host ions. The corrected XRD data in Table 1 show that when the Co concentration increases, the lattice parameters (a = b, c) and unit cell volume (V) decrease. It is worth noting that Co^3+^ ((VI) = 0.565Å) has a lower ionic radius than Ti^4+^ ((VI) = 0.606 Å), but Co^2+^ ((VI) = 0.65Å) has a bigger ionic radius than Ti^4+^ ((VI) = 0.606 Å) [30]. As a result of the reduced unit cell volume, Co^3+^ has mostly doped Ti^4+^ in the BTC lattice. However, because of the valence difference between Co and Ti, Co^3+^ is responsible for including O^2−^ into the lattice. As shown in Figure 2a, the VESTA program was used to depict the t atomic structure of BTC, with Co ions incorporated into the Ti site. This figure shows the oxygen vacancy that develops because of the valence difference between Co ions and Ti. The Scherrer formula, also known as the Scherrer equation, is used in X-ray diffraction (XRD) to estimate the size of crystalline domains within a material based on the width of the fitted (200/002) diffraction peaks in the XRD pattern, such as those in Figure 1b. This formula provides a rough approximation of the average crystallite size in a material, assuming that the broadening of the diffraction peaks with an increase in Co-ion doping is primarily due to the finite size of the crystalline domains.

The Scherrer formula is given by [31]:

(1) $$D_{p}=K\lambda /\beta cos\theta$$

*D* is the average crystallite size in nanometers; *K* is the Scherrer constant, which depends on the shape of the crystalline domains (typically assumed to be around 0.9); *λ* is the wavelength of the X-ray radiation used in nanometers; *β* is the full width at half maximum (FWHM) of the diffraction peak (in radians) obtained from the 200/002 mountains fitting in Figure 1b; and *θ* is the Bragg angle (angle of diffraction) at which the peak occurs (in radians). The obtained crystallite size *D_p_* is plotted in Figure 2c as a function of Co content. The doping of Co ions in the Ti site can lead to the formation of solid solutions, where the Co ions are incorporated into the host lattice. Depending on the charge valency of the Co dopant ions, they may occupy lattice sites that are not necessarily consistent with the ideal crystal structure of the host material. This can lead to lattice distortions and strain, affecting the crystalline lattice size [32]. On the other hand, Co dopants can introduce defects and interfaces in the BT host lattice, such as oxygen vacancies. These defects can act as barriers to crystal growth and affect the coalescence of crystalline domains, potentially leading to smaller crystallite sizes [33].

### 3.2. Raman Analysis

**Figure 3:**
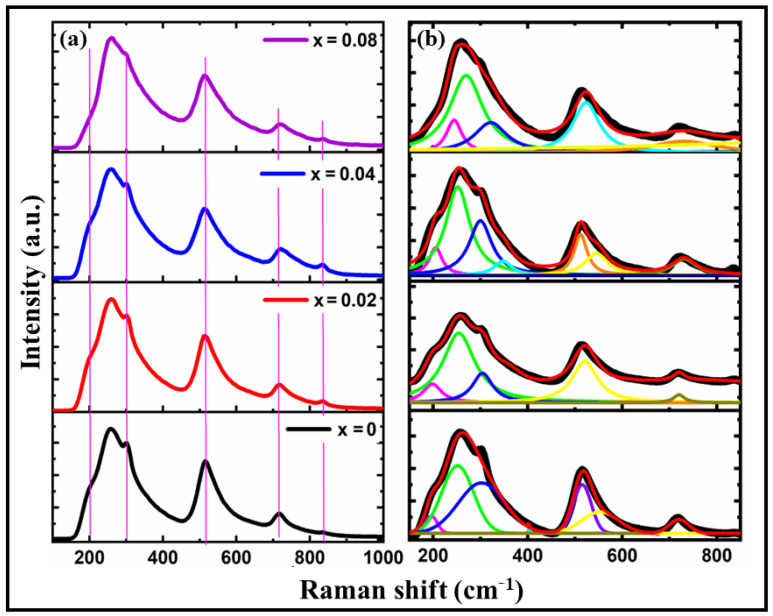
(**a**) Room temperature Raman spectrum, and (**b**) the results of fitting measured via Raman spectra (the thick, solid line represents the fitting curve) of BTCx samples measured using an Nd: YAG laser at 532 nm and a CCD camera.

Raman analysis is a powerful spectroscopic technique used to study a material’s vibrational, rotational, and low-frequency modes. It provides valuable information about molecular vibrations, crystal structures, chemical compositions, etc. Raman spectroscopy is based on the inelastic scattering of light, known as the Raman effect [34,35], where incident photons interact with the sample’s molecules and experience an energy shift. Figure 3 summarizes the Raman spectra of BTCx samples recorded at room temperature in the wavenumber range of 100–1000 cm^−1^. A sharp mode at 305 cm^−1^ [E(LO + TO) + B1] and three broad modes at 262 cm^−1^ E(TO)/[A_1_(TO)], 520 cm^−1^[A_1_(TO_3_)], and 720 cm^−1^ [E(LO)/A_1_(LO)] are present in the tetragonal structure of BaTiO_3_ (BT), as determined by group theory analysis [36]. There is an E(TO_2_) mode at about 199–210 cm^−1^ that is related to the Ba-O vibrations mode; A1(TO_2_), E(TO_3_), and E(LO_3_) modes at about 260–350 cm^−1^ that are associated with the displacement of Co/Ti-O vibrations; and A1(TO_3_) and E(LO_4_) modes that are associated with a combination of bending and stretching in the (Co/Ti)O_6_ octahedral [37,38]. The A_1_g mode is inactive in pure BaTiO_3_ ceramics [39]. According to our findings, the 805–850 cm^−1^ area is devoid of the A1g mode, indicating that it remains a dormant Raman mode for the BT sample. Small A1g Raman peaks within the wavenumber range of 830–836 cm^−1^ were identified as the Co ions incorporated in the Ti site of the BT host lattice. Metal ion inclusion at the Ti-site is confirmed by Raman active modes in this area [40]. A possible explanation for the asymmetric modes at 722 cm^−1^ is that they result from the superposition of (Ti/Co)O_6_ modes. Doping also diminishes the A1(LO_3_) and A1g modes, which may result from significant distortion and cationic disorder [41]. As seen in Figure 3, the octahedral distortion and cationic disorder that appear with metal doping result from B-site ions being randomly dispersed in the center of the deformed octahedron. As illustrated in Figure 3b, the wide bands found in the BTCx spectra due to overlapping modes were unconvoluted using Gaussian fitting. Regarding ionic radius differences, Raman mode shifting may be predicted when an ion with a smaller ionic radius, such as Co^3+^ = 0.565Å-doped Ti^4+^ = 0.605 Å, is present. Thus, it can be stated qualitatively that the octahedral distortion and cationic disorder seen with metal doping are caused by B-site ions randomly dispersed in the center of the octahedron.

### 3.3. Optical Properties

**Figure 4:**
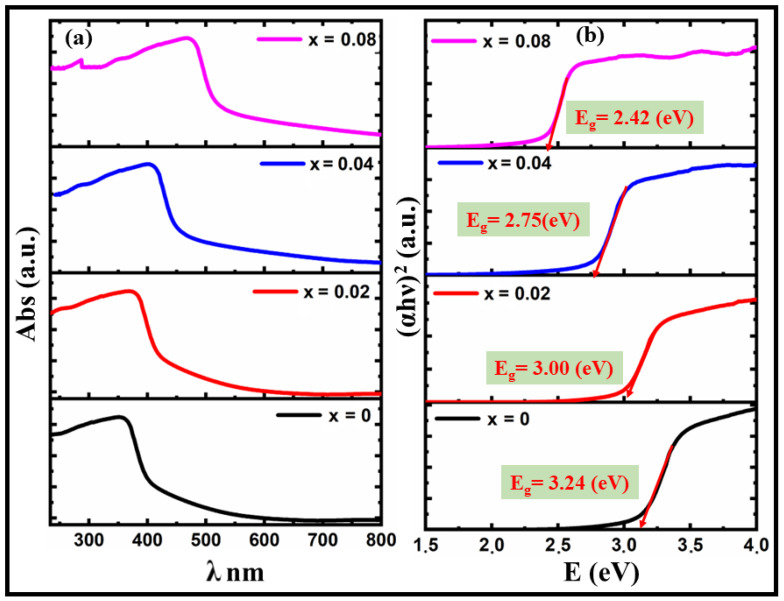
(**a**) UV–vis absorption spectra, and (**b**) Tauc plots of (α*hν*)^2^ versus E for BTCx samples, measured using UV spectroscopy.

The bandgap energy effects of the Co-doped BT matrix were investigated by measuring their optical characteristics. The host ceramic (BT) is expected to have a charge-balanced A-site structure. It is hypothesized that oxygen vacancies are created when dopant Co ions replace Ti^+4^ in the B site of the perovskite structure. The lowest empty orbitals (3d orbitals around Ti atoms) in the conduction band and highest occupied orbitals (2p orbitals around O atoms) in the valence band combine to generate the bandgap in BT samples. Figure 4a displays the observed absorption spectra of BTCx powders between 200 and 800 nm. A Tauc plot is a graphical method used to estimate the bandgap energy of the investigated material based on its absorption spectrum, as can be seen in Figure 4a. The absorption spectrum provides information about light absorption by a material at different wavelengths or energies. From the Tauc plot shown in Figure 4b, the bandgap energies of the BTCx ceramics under study were calculated using the following method [42].

(2) $${\left(\alpha h\upsilon \right)}^{n}=A\left(h\upsilon -E_{g}\right)$$

Here *α* is the absorption coefficient; *hν* is the photon energy (directly related to the wavelength of light); *E_g_* is the bandgap energy of the material; *A* is a constant; and n is the power used to fit the data, often chosen based on the type of electronic transition (e.g., *n* = 1/2 for a direct bandgap and *n* = 2 for an indirect bandgap). The Tauc plot involves plotting the absorption coefficient (*α*) raised to a power (*n*) against the photon energy (*hν*), where α is related to the intensity of light absorbed by the material and *hν* is the energy of the incident photons. The bandgap values are 3.24, 3.00, 2.75, and 2.42 eV for x = 0, 0.02, 0.04, and 0.08, respectively. It is crucial to note that including the Co dopants results in a redshift, as seen in Figure 4. The insertion of metal ions into the BT lattice leads to a smaller bandgap, which suggests the development of new energy levels inside the bandgaps of these ceramics. These energy levels are often the result of octahedral distortion and defect formation, or a combination of the two. This theory provides a straightforward explanation for the observed lower bandgap energy by focusing on the compensatory structural improvements and corrections made by the defect. Introducing Co dopants to the Ti site results in the creation of oxygen vacancies. By considering the different valance states of Co, the charge compensation can be written based on the Kroger–Vink notation as follows [43,44].

(i) Substitution of Co^2+^ at the Ti^4+^ Site with oxygen vacancy compensation:
  (3) $$Co+Ti_{Ti}+O_{o}\to Co_{Ti}^{\prime \prime }+V_{ö}+TiO_{2}$$
(ii) Substitution of M^3+^ at the Ti^4+^ Site with oxygen vacancy compensation:
  (4) $$Co_{2}O_{3}+2Ti_{Ti}+O_{o}\to 2Co_{Ti}^{\prime }+V_{ö}+2TiO_{2}$$

Bandgap energy may be reduced due to those oxygen vacancies via several mechanisms, including structural disorder, defect level creation inside the bandgap, and Fermi-level displacement towards the lowest conduction band [43,44,45,46,47]. These values are very sensitive to the presence of oxygen vacancies. However, as the concentration of oxygen vacancies rises, the bandgap between the most significant defect level and the bottom of the conduction narrows, resulting in more robust absorption spectra and finer tuning of the bandgap. Bandgap reduction due to oxygen-vacancy-induced absorption area overlap. The oxygen-vacancy-compensation-induced defect level moves closer to the conduction band, which interacts with the bottom of the band and pushes it lower.

### 3.4. Band Structure and Density of State Studies

**Figure 5:**
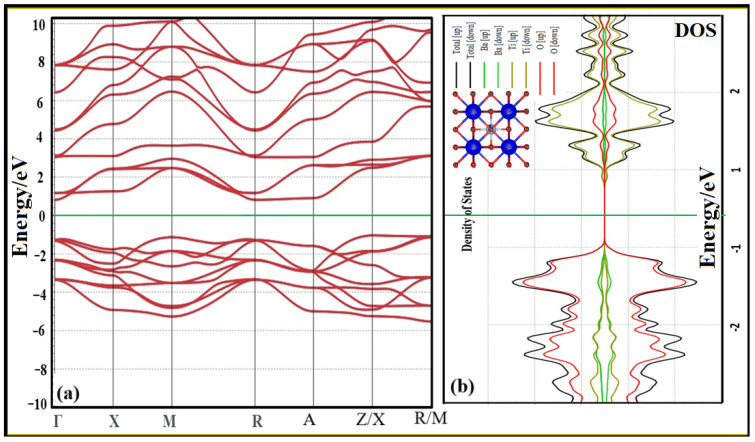
(**a**) The band structure and (**b**) DOS for BTC0.08 systems, carried out based on the data obtained from structural refinement analysis.

Band structure and density of states (DOS) research are crucial in solid-state physics and materials science for understanding the electrical characteristics of materials. These investigations enlighten the electron energy levels’ role in the material’s overall behavior. Figure 5a depicts the band structure of the BTC0.08 sample that was chosen. The energy “bands” that electrons are permitted to inhabit are shown below, illustrating how the band structure of a material represents these limits. This explains how electrons in a crystalline material relate energy and momentum. The bonding type of a material may be inferred from its electronic properties, such as its band structure, density of states (DOS), electron density, etc. The bonding characteristics of the Co-doped BaTiO_3_ sample must be carefully examined. Using the XRD-obtained refinement parameters, we subjected them to DFT analysis to ascertain their electrical properties. To better understand BTC0.08, the electrical band structure in the Brillouin zones in the high symmetry direction is shown in Figure 5a below. The sample has a straight bandgap, meaning an electron may go from the highest energy valence band state to the lowest energy conduction band state without changing the crystal’s momentum. Its relatively large bandgap of 1.84 eV is betrayed by its semiconducting nature. Additional materials should accept the present calculation using this method [48,49,50], since the bandgap of pure BTC0.08 shows excellent consistency with the previously calculated value. This demonstrates a small reduction in the bandgap due to Co doping. The valence and conduction bands of the BTC0.08 sample have distinct peaks near the Fermi level (EF). Furthermore, we found that the hybridization between Ti-3d and O-2p states was most common near the EF. The dominance of the Ti-3d state in the conduction band is analogous to that of the O-ps state.

## 4. Conclusions

Since power conversion efficiencies surpassing the Shockley–Queisser limit were observed in BaTiO_3_ (BTO) crystals, scientific interest in ferroelectric photovoltaics (FPV) involving ferroelectric oxides has increased. However, the advancement in this field has been constrained due to the substantial bandgap (>3 eV) characteristics of ferroelectric oxides. In response, this investigation introduces and presents a novel ferroelectric BaTi_1−x_Co_x_O_3_ powder (abbreviated as BTCx) with varying Co content (0 ≤ x ≤ 0.08), exhibiting a notable reduction in bandgap as the Co content increases. Of particular significance, the alteration in composition from x = 0.0 to 0.08 triggered a pronounced decline in the bandgap, which transitions from 3.24 to 2.42 eV. The most remarkable outcome was observed in the optimized configuration at x = 0.08, demonstrating an unconventional photovoltaic response. Employing Raman spectroscopy, the study delved into the origins of this bandgap reduction. The application of density functional theory lends insight into interpreting the experimental findings. According to our investigations, it is apparent that Co doping and perturbations in the oxygen octahedral structure synergistically contribute to the effective reduction of the bandgap. This research not only elucidates the mechanisms underlying the development of bandgap modulation but also paves the way for exploring innovative ferroelectric materials with low bandgaps, holding significant promise for advancing next-generation photovoltaic applications.

## Figures and Tables

**Table 1:** Parameters obtained from Rietveld refinement for BTCx samples.

Sample/Parameter	x = 0	x = 0.02	x = 0.04	x = 0.08
Crystal System	Tetragonal	Tetragonal	Tetragonal	Tetragonal
a = b (Å)	3.9943 ± 0.001	3.9928 ± 0.001	3.9913 ± 0.001	3.9899 ± 0.001
c (Å)	4.0267 ± 0.001	4.0154 ± 0.001	4.0137 ± 0.001	4.0125 ± 0.001
V (Å)^3^	64.2104 ± 0.001	64.0147 ± 0.001	63.9386 ± 0.001	63.8762 ± 0.001
c/a	1.0084 ± 0.001	1.0057 ± 0.001	1.00562 ± 0.001	1.00561 ± 0.001
Space group	P 4 mm	P 4 mm	P 4 mm	P 4 mm
Density g/cm^3^	6.058	6.047	6.024	6.011
Crystallite size (nm)	37	33	30	28
Lattice strain	0.0025	0.0027	0.0031	0.0033
R_p_ (%)	4.71	6.42	5.83	6.75
R_wp_ (%)	5.55	5.72	6.98	8.31
R_ex_ (%)	5.33	5.17	5.47	5.83
χ^2^	1.08	1.22	1.62	2.03

## Data Availability

Not applicable.
